# Supplementary material for: Maximal Intensity Exercise Induces Adipokine Secretion and Disrupts Prooxidant–Antioxidant Balance in Young Men with Different Body Composition
Source: Int J Mol Sci. 2025 Jan 3;26(1):350. doi: 10.3390/ijms26010350 (PMC11721682; doi:10.3390/ijms26010350)
Supplement: Supplementary file 1 [file ijms-26-00350-s001.zip › ijms-3315047-supplementary.pdf]

Supplementary Materials

Table S1. Concentration of prooxidant-antioxidant balance markers before (T0) and after maximal intensity exercise without correction (T1) and after correction for plasma volume changes ($T1_{PV}$)

		NFAT-NLBM	NFAT-HLBM	HFAT-NLBM
**VARIABLE**		**Mean±SD**	**Mean±SD**	**Mean±SD**
AOPP	T0	25.94±9.67	24.50±3.66	26.72±4.60
(μmol×L^{-1})	T1	27.41±7.15	30.56±5.71	30.48±5.06
	$T1_{PV}$	25.43±6.89	27.30±5.31	27.14±5.01
DNA/RNA/ox	T0	2.06±2.15	3.53±3.77	2.50±2.41
(ng×mL^{-1})	T1	3.01±3.84	4.09±5.02	2.66±2.42
	$T1_{PV}$	2.83±3.71	3.68±4.51	2.42±2.30
LPO	T0	163.85±73.3	208.89±90.52	177.73±76.70
(μmol×L^{-1})	T1	214.48±80.61	258.88±106.80	243.12±103.62
	$T1_{PV}$	198.02±71.35	231.02±95.20	217.60±96.47
CAT	T0	38.27±17.65	46.02±12.04	38.16±16.02
(nmol×min^{-1}×mL^{-1})	T1	51.32±16.30	54.56±21.64	59.81±18.32
	$T1_{PV}$	47.66±15.55	48.30±17.14	53.01±15.45
SOD	T0	13.11±3.33	13.90±2.50	13.99±4.11
(μmol×min^{-1}×mL^{-1})	T1	13.04±3.72	13.89±2.12	13.37±2.71
	$T1_{PV}$	12.07±3.30	12.46±2.25	11.91±2.64
GPx	T0	48.37±18.25	57.41±19.58	57.73±19.15
(nmol×min^{-1}×mL^{-1})	T1	58.12±18.92	59.31±27.37	69.35±33.45
	$T1_{PV}$	53.84±17.30	52.70±23.89	61.89±30.31

AOPP - advanced protein oxidation products; DNA/RNA/ox - markers of oxidative damage to nucleic acids DNA and RNA; LPO - markers of lipid peroxidation; CAT - catalase; SOD - superoxide dismutase; GPx - glutathione peroxidase; NFAT-NLBM - group with average content of %FAT and with average content of LBM, NFAT-HLBM - group with average content of %FAT and above-average content of LBM, HFAT-NLBM – group with above-average content of %FAT and average content of LBM.

Table S2. Post-exercise changes in concentration of prooxidant-antioxidant balance markers

VARIABLE		Mean (95%CI) NFAT-NLBM	Mean (95%CI) NFAT-HLBM	Mean (95%CI) HFAT-NLBM	Statistical analysis *p*-value (*post-hoc* Tukey's HSD test) NFAT-NLBM	NFAT-HLBM	HFAT-NLBM
AOPP	T1-T0	1.48 (-1.70-4.65)	6.06 (3.84-8.28)	3.76 (0.90-6.62)	0.86	**<0.01**	0.06
(μmol×L^{-1})	$T1_{PV}$-T0	-0.50 (-3.78-2.77)	2.80 (0.77-4.83)	0.42 (-2.04-2.89)	NS ANOVA	NS ANOVA	NS ANOVA
DNA/RNA/ox	T1-T0	0.96 (-0.60-2.52)	0.55 (-0.41-1.52)	0.16 (-0.67-0.98)	NS ANOVA	NS ANOVA	NS ANOVA
(ng×mL^{-1})	$T1_{PV}$-T0	0.78 (-0.70-2.26)	0.15 (-0.63-0.92)	-0.08 (-0.94-0.78)	NS ANOVA	NS ANOVA	NS ANOVA
LPO	T1-T0	50.63 (31.97-69.30)	49.99 (25.72-74.26)	65.40 (39.34-91.45)	<0.01	<0.01	<0.01
(μmol×L^{-1})	$T1_{PV}$-T0	34.17 (14.09-54.24)	22.13 (0.40-43.86)	39.87 (15.28-64.46)	0.03	0.049	0.01
CAT	T1-T0	13.05 (1.88-24.22)	8.54 (-0.32-17.40)	21.65 (7.96-35.5)	0.17	0.49	<0.01
(nmol×min^{-1}×mL^{-1})	$T1_{PV}$-T0	9.39 (-1.81-20.59)	2.29 (-4.95-9.52)	14.85 (1.97-27.73)	0.42	0.99	0.048
SOD	T1-T0	-0.07 (-1.31-1.18)	-0.01(-1.35-1.34)	-0.62 (-2.18-0.95)	NS ANOVA	NS ANOVA	NS ANOVA
(μmol×min^{-1}×mL^{-1})	$T1_{PV}$-T0	-1.04 (-2.13-0.05)	-1.44 (-2.90-0.01)	-2.07 (-3.73-(-0.41))	0.67	0.21	0.049
GPx	T1-T0	9.75 (-3.50-23.01)	1.90 (-6.37-10.18)	11.62 (-9.81-33.06)	0.72	0.99	0.56
(nmol×min^{-1}×mL^{-1})	$T1_{PV}$-T0	5.47 (-6.72-17.66)	-4.71 (-11.97-2.55)	4.16 (-16.22-24.54)	NS ANOVA	NS ANOVA	NS ANOVA

AOPP - advanced protein oxidation products; DNA/RNA/ox - markers of oxidative damage to nucleic acids DNA and RNA; LPO - markers of lipid peroxidation; CAT - catalase; SOD - superoxide dismutase; GPx - glutathione peroxidase; level before (T0) and after maximal intensity exercise without correction (T1) and after correction for plasma volume changes ($T1_{PV}$); CI - confidence interval; $p<0.05$ - statistically significant difference T1 vs. T0 and $T1_{PV}$ vs. T0 in each group (*post-hoc* Tukey's HSD test following two-way repeated measures analysis of variance for data (1) without correction for %dPV of post-exercise values and independently (2) with correction for %dPV of post-exercise values); NS ANOVA - no significant effect of the main factor: EXERCISE (p>0.05) - no post-hoc analysis; NFAT-NLBM - group with average content of %FAT and with average content of LBM, NFAT-HLBM - group with average content of %FAT and above-average content of LBM, HFAT-NLBM – group with above-average content of %FAT and average content of LBM.

Table S3. Concentration of adipokines before (T0) and after maximal intensity exercise without correction (T1) and after correction for plasma volume changes ($T1_{PV}$)

		NFAT-NLBM	NFAT-HLBM	HFAT-NLBM
**VARIABLE**		**Mean±SD**	**Mean±SD**	**Mean±SD**
Visfatin	T0	0.87±1.13	0.45±0.46	1.15±2.73
(ng×mL^{-1})	T1	1.72±1.85	0.87±0.55	1.53±2.90
	$T1_{PV}$	1.59±1.72	0.77±0.46	1.27±2.22
Leptin	T0	2.54±0.76	2.60±0.90	5.61±6.42
(ng×mL^{-1})	T1	2.84±0.99	2.87±1.09	6.35±7.57
	$T1_{PV}$	2.63±0.93	2.57±0.02	5.79±7.20
Resistin	T0	4.41±1.10	4.30±1.15	3.72±1.05
(ng×mL^{-1})	T1	5.74±1.40	5.78±1.38	5.19±1.54
	$T1_{PV}$	5.33±1.30	5.18±1.31	4.60±1.32
Adiponectin	T0	11.32±2.00	11.47±2.52	10.32±1.52
(µg×mL^{-1})	T1	12.27±2.01	12.27±2.67	11.23±1.54
	$T1_{PV}$	11.41±2.16	10.99±2.58	9.95±1.07
Asprosin	T0	16.24±16.65	12.56±10.13	14.60±10.93
(ng×mL^{-1})	T1	13.94±15.67	12.44±9.60	12.55±6.15
	$T1_{PV}$	12.97±14.7	11.17±8.72	13.48±10.09
Irisin	T0	1.48±0.42	1.59±0.91	1.46±0.49
(µg×mL^{-1})	T1	1.85±0.65	1.77±0.81	1.71±0.52
	$T1_{PV}$	1.73±0.66	0.58±0.68	1.53±0.50

NFAT-NLBM - group with average content of %FAT and with average content of LBM, NFAT-HLBM - group with average content of %FAT and above-average content of LBM, HFAT-NLBM – group with above-average content of %FAT and average content of LBM.

Table S4. Post-exercise changes in adipokine concentration

VARIABLE		Mean (95%CI) NFAT-NLBM	NFAT-HLBM	HFAT-NLBM	Statistical analysis *p*-value (*post-hoc* Tukey's HSD test) NFAT-NLBM	NFAT-HLBM	HFAT-NLBM
Visfatin	T1-T0	0.86 (0.28-1.43)	0.41 (0.18-0.65)	0.38 (0.14-0.62)	<0.01	0.12	0.27
(ng×mL^{-1})	T1$_{PV}$-T0	0.72 (0.24-1.21)	0.32 (0.12-.51)	0.12 (-0.25-0.50)	<0.01	0.31	0.98
Leptin	T1-T0	0.29 (0.09-0.49)	0.27 (0.15-0.38)	0.74 (-0.02-1.50)	0.71	0.69	0.01
(ng×mL^{-1})	T1$_{PV}$-T0	0.08 (-0.11-0.28)	-0.03 (-0.12-0.05)	0.18 (-0.41-0.76)	NS ANOVA	NS ANOVA	NS ANOVA
Resistin	T1-T0	1.33 (0.98-1.68)	1.48 (1.24-1.72)	1.47 (0.99-1.95)	<0.01	<0.01	<0.01
(ng×mL^{-1})	T1$_{PV}$-T0	0.92 (0.53-1.31)	0.88 (0.67-1.10)	0.88 (0.48-1.28)	<0.01	0.02	<0.01
Adiponectin	T1-T0	0.94 (0.28-1.61)	0.80 (0.35-1.25)	0.91 (0.42-1.39)	0.01	0.01	0.01
(μg×mL^{-1})	T1$_{PV}$-T0	0.08 (0.77-0.94)	-0.48 (-1.00-0.04)	-0.37 (-1.07-0.33)	NS ANOVA	NS ANOVA	NS ANOVA
Asprosin	T1-T0	-2.30 (-4.90-0.29)	-0.11 (-1.81-1.58)	0.28 (-3.80-4.35)	NS ANOVA	NS ANOVA	NS ANOVA
(ng×mL^{-1})	T1$_{PV}$-T0	-3.27(-6.01-(-0.53))	-1.39 (-3.10-0.32)	-1.12 (-5.21-2.97)	0.19	0.87	0.96
Irisin	T1-T0	0.37 (0.07-0.67)	0.19 (-0.14-0.51)	0.24 (0.00-0.48)	0.12	0.70	0.54
(μg×mL^{-1})	T1$_{PV}$-T0	0.25 (-0.06-0.55)	-0.01 (-0.34-0.32)	0.06 (-0.19-0.31)	NS ANOVA	NS ANOVA	NS ANOVA

Level before (T0) and after maximal intensity exercise without correction (T1) and after correction for plasma volume changes (T1$_{PV}$). CI - confidence interval; $p<0.05$ - statistically significant difference T1 vs. T0 and T1$_{PV}$ vs. T0 in each group (*post-hoc* Tukey's HSD test following two-way repeated measures analysis of variance for data (1) without correction for %dPV of post-exercise values and independently (2) with correction for %dPV of post-exercise values); NS ANOVA - no significant effect of the main factor: EXERCISE (p>0.05) - no post-hoc analysis; NFAT-NLBM - group with average content of %FAT and with average content of LBM, NFAT-HLBM - group with average content of %FAT and above-average content of LBM, HFAT-NLBM – group with above-average content of %FAT and average content of LBM.
